# Supplementary material for: Validation of a Questionnaire to Assess Smoking Habits, Attitudes, Knowledge, and Needs among University Students: A Pilot Study among Obstetrics Students
Source: Int J Environ Res Public Health. 2021 Nov 12;18(22):11873. doi: 10.3390/ijerph182211873 (PMC8621372; doi:10.3390/ijerph182211873)
Supplement: Supplementary file 1 [file ijerph-18-11873-s001.zip › ijerph-1434354-supplementary.pdf]

Supplementary files

Validation of a questionnaire to assess smoking habits, attitudes, knowledge, and needs among university students: a pilot study among obstetrics students

Laura Campo[1,2], Francesca Vecera[2], Silvia Fustinoni[1,2]

[1]Environmental and Industrial Toxicology Unit, Fondazione IRCCS Ca' Granda Ospedale Maggiore Policlinico, Milan, Italy

[2]EPIGET - Epidemiology, Epigenetics, and Toxicology Lab, Department of Clinical Sciences and Community Health, Università degli Studi di Milano, Italy

Table S1. Section A: Study participants (89 responders).

Question, statistics	Anchors	Response
sex, N (%)	Male	3 (3%)
	Female	86 (97%)
	Intersex	0
	I'd rather not answer	0
age (years), mean (min-max)		22 (19-38)
Which is your course year, N (%)	1° course year	34 (75%)
	2° course year	27 (73%)
	3° course year	26 (81%)
Do you currently smoke? N (%)	No, i.e. I have never smoked or have smoked less than 100 traditional cigarettes (commercial or hand-rolled) in my life *(go to sections C - D - E - F - G)*	64 (72%)
	No, but I used to, i.e. I have smoked at least 100 traditional cigarettes (commercial or hand-rolled) in my life *(go to sections C - D - E - F - G)*	4 (4%)
	Yes, I smoke traditional cigarettes only (commercial or hand-rolled) *(go to sections B - D - E - F - G - H)*	16 (18%)
	Yes, I use electronic cigarettes (e-cigs) or heated tobacco products (HTPs) only *(go to sections C - D - E - F - G)*	4 (5%)
	yes, I smoke both traditional cigarettes and e-cigs / HTPs *(go to sections B - D - E - F - G)*	1 (1%)

Table S2. Section B: Active smoking of traditional tobacco cigarettes (commercial or hand-rolled) (17 responders: 16 exclusively cigarette smokers and 1 dual smoker).

Question, statistics	Anchors	Response
1) At what age did you start smoking traditional cigarettes?<br>age (years), mean (min-max)		17 (14-20)
2) Do you remember why you started smoking traditional cigarettes? (You can tick more than one box.)<br>N (%)	Peer pressure / at parties / my friends smoked	13 (76%)
	Because I tried and I liked it / it gave me pleasure	10 (59%)
	Grown-up feeling / sense of empowerment	1 (5,9%)
	Influence from family / there were smokers in my family	2 (12%)
	Influence from my partner	1 (6%)
	Because I was feeling anxious / stressed	7 (41%)
	Because it made me feel confident	3 (18%)
	Other, specify_______________	0
3) How often do you currently smoke?<br>N (%)	Not every day *(go to question 3a)*	9 (53%)
	Every day *(go to question 3b)*	8 (47%)
3a) How many traditional cigarettes do you smoke per week?<br>N (%)	1-4	4 (24%)
	5-9	4 (24%)
	10-14	1 (6%)
	15-19	0
	≥ di 20	0
3b) How many traditional cigarettes do you smoke per day?<br>N (%)	1-4	4 (24%)
	5-9	2 (12%)
	10-14	1 (6%)
	15-19	1 (6%)
	≥ di 20	0
4) Where do you usually smoke traditional cigarettes?<br>N (%)	Only outdoors	15(88%)
	Only indoors (e.g. home, car, smoking areas of public places)	0
	Both indoors and outdoors	2(12%)
5) Where do you smoke traditional cigarettes on campus? (You can tick more than one box.)<br>N (%)	I do not smoke	5 (29%)
	Outdoors (e.g. courtyard, balconies, external stairs, porches)	8 (47%)
	In smoking areas	7 (41%)
	Indoors (e.g. hallways, washrooms, study areas)	0
6) When you are with your family, you smoke traditional cigarettes:<br>N (%)	openly	8 (47%)
	secretly	9 (53%)
7) Have you ever tried to quit smoking traditional cigarettes?<br>N (%)	Yes	10 (59%)
	No *(go to question 9)*	7 (41%)
8) How long have you abstained from smoking traditional cigarettes, at most?<br>N (%)	< 7 days	0
	8 days-30 days	1 (6%)
	> 30 days-6 months	7 (41%)
	> 6 months-1 year	1 (6%)
	> 1 year	0
9) Have you ever been advised by a doctor or other healthcare professional to quit smoking traditional cigarettes?<br>N (%)	yes	10 (59%)
	No	5 (29%)
	I do not remember	2 (12%)
10) Are you planning to quit smoking traditional cigarettes in the next six months?<br>N (%)	Yes	5 (29%)
	No	6 (35%)
	I don't know	6 (35%)
**Fagerström test**		
1) How long after you wake up do you smoke your first cigarette?<br>N (%)	Within 5 minutes	1 (6%)
	6 to 30 minutes	1 (6%)
	31 to 60 minutes	0
	After 60 minutes	15 (88%)
2) Do you find it difficult to refrain from smoking in placesa where it is forbidden?<br>N (%)	Yes	0
	No	17 (100%)
3) Which cigarette would you hate most to give up?	First cigarette in the morning	2 (12%)

N (%)	All other cigarettes	15 (88%)
4) How many cigarettes do you smoke per day?<br>N (%)	10 or less	16 (94%)
	11-20	1 (5,9%)
	21-30	0
	31 or more	0
5) Do you smoke more frequently during the first hour after waking up than during the rest of the day?<br>N (%)	Yes	1 (6%)
	No	16 (94%)
6) Do you smoke even if you are so ill that you are in bed most of the day?<br>N (%)	Yes	2 (12%)
	No	15 (88%)

Table S3. Section C: Former smoker of traditional tobacco cigarettes (4 responders).

Question, statistics	Anchors	Response
1) Have you smoked at least 100 traditional cigarettes (commercial or hand-rolled) in your life?<br>N (%)	Yes	4 (4%)
	No *(go to section D)*	85 (96%)
2) At what age did you start smoking traditional cigarettes?<br>Years, mean (min-max)		16 (15-17)
3) How many traditional cigarettes did you smoke per day?<br>mean (min-max)		5 (3-10)
4) How long have you been without smoking traditional cigarettes?	< 7 days	0
	8 days-30 days	0
	> 30 days-6 months	0
	>6 months-1year	1 (25%)
	>1 yeasr	3 (75%)
5) Do you remember why you started smoking traditional cigarettes? (You can tick more than one box)<br>N (%)	Peer pressure / at parties / my friends smoked	1 (25%)
	Because I tried and I liked it / it gave me pleasure	2 (50%)
	Grown-up feeling / sense of empowerment	1 (25%)
	Influence from family / there were smokers in my family	0
	Influence from my partner	0
	Because I was feeling anxious / stressed	3 (75%)
	Because it made me feel confident	1 (25%)
	Other, specify_______________	0
6) How did you manage to quit smoking traditional cigarettes? (You can tick more than one box)<br>N(%)	Unconventional therapies (e.g. acupuncture phytotherapy, etc.)	0
	Support groups	0
	Nicotine replacement therapy (e.g. patches, chewing gum, tablets, etc.)	0
	Alone, without help	4 (100%)
	With the help of a doctor or other healthcare professional	0
	Smoke-free advice centre	0
	Smoke-free apps (on smartphones / tablets)	0
	Other, specify_______________	0
7) Why did you quit smoking traditional cigarettes? (You can tick more than one box.)<br>N(%)	Health concern	3 (75%)
	Concern for the health of those around me	0
	To save money	0
	To feel psychologically free	2 (50%)
	To improve my looks	0
	I did not like it	1 (25%)
	Other, specify_______________	0

Table S4. Section D: Electronic cigarettes or HNB users (3 responders to items D2-D9, 13 responders to items D10-D11, 89 to items D1 and D12, 5 responders to item D13-D19, 11 responders to items D20-D21).

Question, statistics	Anchors	Response
1) Have you used electronic cigarettes (e-cigs) in the last week?<br>N (%)	Yes	3 (3%)
	No, but I did in the past *(go to question D10)*	10 (11%)
	No, never have *(go to question D12)*	76 (85%)
2) How long have you been using e-cigs?	< 7 days	0
	8 days-30 days	0
	31 days-6 months	3 (100%)
	> 6 months-1 year	0
	> 1 year	0
3) What kind of e-liquids do you mostly use? (You can tick more than one box)<br>N (%)	With nicotine, no flavours	2 (67%)
	With nicotine and flavours	0
	No nicotine, with flavours	1 (33%)
4) How many ml of liquids do you use per day?<br>N (%)	<2	0
	>2-3	0
	≥ 4	3 (100%)
5) Where do you usually use e-cigs?<br>N (%)	Only outdoors	2 (67%)
	Only indoors (e.g. home, car, smoking areas of public places)	0
	Both indoors and outdoors	1 (33%)
6) Where do you use your e-cig on campus? (You can tick more than one box)<br>N (%)	I do not use it	1 (33%)
	Outdoors (e.g. courtyard, balconies, external stairs, porches)	1 (33%)
	In smoking areas	1 (33%)
	Indoors (e.g. hallways, washrooms, study areas)	0
7) When you are with your family, you use e-cigs:<br>N (%)	Openly	2 (67%)
	Secretly	1 (33%)
8) Have you ever been advised by a doctor or other healthcare professional to quit e-cigs?N (%)	Yes	1 (33%)
	No	2 (67%)
	I do not remember	0
9) Are you going to stop using e-cigs in the next six months?<br>N (%)	Yes	2 (67%)
	No	1 (33%)
	I don't know	0
10) Why did you start using e-cigs? (You can tick more than one box)<br>N (%)	I think it is less dangerous to my health as compared to smoking traditional cigarettes	3 (23%)
	As an alternative to traditional cigarettes	5 (38%)
	Trendy / out of curiosity	3 (23%)
	So that I could smoke where it is forbidden to smoke traditional cigarettes	0
	As an aid to quit traditional cigarettes	5 (38%)
	Other, specify_______________	1 (8%)
11) After you started using e-cigs:<br>N (%)	I have given up traditional cigarettes	4 (31%)
	I have started or gone back to smoking traditional cigarettes	6 (46%)
	I have been smoking less traditional cigarettes	2 (15%)
	I have been smoking more traditional cigarettes	0
	I did not smoke and still do not smoke traditional cigarettes	1 (8%)
12) Have you used HTPs in the last week (e.g.. IQOS)?<br>89	Yes	5 (6%)
	No, but I did in the past (go to question 20)	6 (7%)
	No, never have (go to Section E)	78 (88%)
13) How long have you been using HTPs?<br>N (%)	< 7 days	0
	8 days-30 days	0
	31 days-6 months	2 (40%)
	> 6 months-1 year	2 (40%)
	> 1 year	1 (20%)
14) How many refills do you use per day?<br>N (%)	< 1 -4	0
	5-10	2 (40%)
	11-15	3 (60%)
	≥ 16	0
15) Where do you usually use HTPs?	Only outdoors	1 (20%)

N (%)	Only indoors (e.g. home, car, smoking areas of public places)	0
	Both indoors and outdoors	4 (80%)
16) Where do you use your HTPs on campus? (You can tick more than one box)<br>N (%)	I do not use it	1 (20%)
	Outdoors (e.g. courtyard, balconies, external stairs, porches)	2 (40%)
	In smoking areas	3 (60%)
	Indoors (e.g. hallways, washrooms, study areas)	0
17) When you are with your family, you use HTPs<br>N (%)	Openly	3 (60%)
	Secretly	2 (40%)
18) Have you ever been advised by a doctor or healthcare professional to quit HTPs?<br>N (%)	Yes	1 (20%)
	No	4 (80%)
	I do not remember	0
19) Are you going to quit HTPs in the next six months?<br>N (%)	Yes	1 (20%)
	No	1 (20%)
	I don't know	3 (60%)
20) Why did you start using HTPs? (You can tick more than one box)<br>N (%)	I think it is less dangerous to my health as compared to smoking traditional cigarettes	4 (36%)
	As an alternative to traditional cigarettes	6 (55%)
	Trendy / out of curiosity	3 (27%)
	So that I could smoke where it is forbidden to smoke traditional cigarettes	0
	As an aid to quit traditional cigarettes	1 (9%)
	Other, specify________________	
21) After you started using HTPs:<br>N (%)	I have given up traditional cigarettes	5 (45%)
	I have started or gone back to smoking traditional cigarettes	4 (36%)
	I have been smoking less traditional cigarettes	0
	I have been smoking more traditional cigarettes	0
	I did not smoke and still do not smoke traditional cigarettes	2 (18%)

Table S5. Section E: Passive smoking (89 responders)

Question, statistics	Anchors		Response
1) Do you live with any smokers?<br>N (%)	Yes, and they smoke in my presence / at home		20 (22%)
	Specify product(s) (You can tick more than one box.):	traditional cigarettes	19 (95%)
		e-cigs or HTPs	3 (15%)
		other (pipe / cigar)	0
	Yes, but they do not smoke in my presence / at home		19 (21%)
	No		50 (56%)
2) Do you usually spend leisure time with smokers?<br>N (%)	Yes, and they smoke in my presence		51 (57%)
	Specify product(s) (You can tick more than one box.):	traditional cigarettes	49 (96%)
		e-cigs or HTPs	22 (43%)
		other (pipe / cigar)	1 (2%)
	Yes, but they do not smoke in my presence		13 (15%)
	No		25 (28%)
3) Over the last week, have you been exposed to passive smoking continuously (for at least 10 minutes)?<br>N (%)	Yes		27 (30%)
	No *(go to question 5)*		62 (70%)
4) Over the last week, where have you been exposed to passive smoking continuously (for at least 10 minutes)?<br>N (%)	Outdoors *(go to question 4A)*		17 (63%)
	Indoors *(go to question 4B and then 4C)*		5 (19%)
	Both indoors and outdoors *(go to question 4B and then 4C)*		5 (19%)
4A) which product were you exposed to? (You can tick more than one box)<br>N (%)	Traditional cigarettes		16 (94%)
	E-cigs or HTPs		7 (41%)
	Other (pipe / cigar)		0
4B) Over the last week, where have you been exposed to passive smoking indoors? (You can tick more than one box.)<br>N (%)	At home		7 (70%)
	In the car		0
	Other (public places, friends' houses, etc.)		5 (50%)
4C) Which product were you exposed to? (You can tick more than one box.)<br>N (%)	Traditional cigarettes		10 (100%)
	E-cigs or HTPs		3 (30%)
	Other (pipe / cigar)		0
5) In your house, traditional cigarettes:<br>N (%)	Are not allowed in any rooms		42 (47%)
	Are only allowed in some rooms		6 (7%)
	Are only allowed outdoors (e.g. balcony / terrace / garden)		37 (42%)
	Are allowed everywhere		4 (4,4%)
6) In your house, e-cigs and HTPs:<br>N (%)	Are not allowed in any rooms		43 (48%)
	Are only allowed in some rooms		8 (9%)
	Are only allowed outdoors (e.g. balcony / terrace / garden)		30 (34%)
	Are allowed everywhere		8 (9%)

Table S6. Section F: Knowledge of smoking health related issues and role of healthcare professionals (89 responders).

Question, statistics	Anchors	Response
1) Is active cigarette smoking bad for your health? N (%)	Yes	89 (100%)
	Yes, but only in particular conditions (e.g. illness, pregnancy)	0
	No	0
	I don't know	0
2) Is active e-cig smoking bad for your health? N (%)	Yes	76 (85%)
	Yes, but only in particular conditions (e.g. illness, pregnancy)	2 (2%)
	No	0
	I don't know	11 (12%)
3) Is active HTP smoking bad for your health? N (%)	Yes	79 (89%)
	Yes, but only in particular conditions (e.g. illness, pregnancy)	0
	No	0
	I don't know	10 (11%)
4) E-cigs and HTPs, as compared to traditional cigarettes, are: N (%)	More harmful to the health of smokers	2 (2%)
	Equally harmful to the health of smokers	35 (39%)
	Less harmful to the health of smokers	33 (37%)
	I don't know	19 (21%)
5) Do you think passive smoking from traditional cigarettes is harmful to the health of non-smokers? N (%)	Yes	84 (94%)
	Yes, but only in particular conditions (e.g. illness, pregnancy)	4 (4%)
	No	0
	I don't know	1 (1%)
6) Do you think passive smoking from e-cigs or HTPs is harmful to the health of non-smokers? N (%)	Yes	68 (76%)
	Yes, but only in particular conditions (e.g. illness, pregnancy)	5 (6%)
	No	4 (4%)
	I don't know	12 (13%)
7) What is the appeal of e-cigs and HTPs in your opinion? (You can tick more than one box) N (%)	They do not leave a persistent smell	42 (47%)
	I think active smoking of these products is less dangerous to your health than traditional cigarette smoking	27 (30%)
	I think passive smoking of these products is less dangerous to your health than traditional cigarette smoking	18 (20%)
	They may be used where traditional cigarettes cannot be smoked	38 (43%)
	You have different flavours	43 (48%)
	They are trendy	3 (3%)
	I am not familiar with these products	9 (10%)
	I don't know	10 (11%)
	Other, specify_______________	3 (3%)
8) What are negative features of e-cigs and HTPS in your opinion? (You can tick more than one box) N (%)	They are too expensive	21 (24%)
	There is no certainty about the long-term health effects of these products	48 (54%)
	They may lead non-smokers to smoke	51 (57%)
	They are difficult to find	1 (1%)
	They require maintenance / must be handled carefully	15 (17%)
	I am not familiar with these products	9 (10%)
	I don't know	13 (15%)
	Other, specify_______________	1 (1%)
9) Which initiatives can help quit smoking or prevent young people from starting smoking in your opinion? (You can tick more than one box) N (%)	Increase in prices	38 (43%)
	Informative advertising on the harm of smoking	43 (48%)
	More information in schools on the harm of smoking	17 (19%)
	Ban on smoking on TV / in movies	28 (31%)
	Extension of the smoking ban to outdoors (e.g. parks / gardens / bus stops)	46 (52%)
	None of these	6 (7%)
	Other, specify_______________	4 (4%)
	I don't know	9 (10%)
10) Do healthcare professionals serve as role models for their patients and society in terms of smoking habits? N (%)	Yes	63 (71%)
	No	15 (17%)
	I don't know	11 (12%)
11) Should healthcare professionals routinely advise	Yes	80 (90%)

their patients who smoke traditional cigarettes to quit smoking?<br>N (%)	No	2 (2%)
	I don't know	7 (8%)
12) Should healthcare professionals regularly advise their patients who use e-cigs and / or HTPs to stop using these products?<br>N (%)	Yes	76 (85%)
	No	2 (2%)
	I don't know	11 (12%)
13) Should healthcare professionals receive specific training on smoking cessation techniques?<br>N (%)	Yes	66 (74%)
	No	8 (9%)
	I don't know	15 (17%)
14) Do healthcare professionals have a role in giving advice or information on smoking cessation to patients?<br>N (%)	Yes	77 (87%)
	No	3 (3%)
	I don't know	9 (10%)
15) Are healthcare professionals who smoke traditional cigarettes less likely to advise patients to quit smoking?<br>N (%)	Yes	43 (48%)
	No	21 (24%)
	I don't know	25 (28%)
16) Are healthcare professionals who use other products (e-cigs and / or HTPs) less likely to advise patients to quit smoking?<br>N (%)	Yes	38 (43%)
	No	19 (21%)
	I don't know	32 (36%)
17) During your university studies, did you learn that it is important to record the history of tobacco use as part of a patient's general medical history?<br>N (%)	Yes	81 (91%)
	No	2 (2%)
	I don't know	6 (7%)
18) During your university studies, have you ever received any formal training in smoking cessation approaches to be used with patients?<br>N (%)	Yes	20 (22%)
	No	64 (72%)
	I don't know	5 (6%)
19) During which university course, did you learn about the effects of cigarette smoking on health? (You can tick more than one box.)<br>N (%)	During my Bachelor's degree programme	61 (69%)
	During my Master's or single-cycle degree programme	2 (2%)
	Never	17 (19%)
	I don't know	14 (16%)
20) On which of the following topics concerning cigarette smoking would you be interested in receiving more information during your university studies? (You can tick more than one box.)<br>N (%)	Health effects of traditional cigarette smoking	34 (38%)
	Smoking cessation techniques	58 (65%)
	The dangers of passive smoking	49 (55%)
	Health effects of new products (e-cigs and / or HTPs)	59 (66%)
	Other, specify_______________	1 (1%)
	I find my training on these topics satisfactory	7 (8%)

Table S7. Section G: Knowledge and attitudes towards Italian smoking legislation, and educational needs (89 responders).

Question, statistics	Anchors	Response
1) To protect the health of non-smokers, the law prohibits smoking indoors in public places, e.g. restaurants, trains, etc. (Law no. 3 of 2003, art. 51). Do you think the law serves its purpose? N (%)	Yes	84 (94%)
	No	4 (4)
	I don't know	1 (1%)
2) Are shock images on cigarette packs effective as a health warning? N (%)	Yes	12 (13%)
	No	68 (76%)
	I don't know	9 (10%)
3) Are you aware that it is forbidden to smoke in vehicles in the presence of children and pregnant women? N (%)	Yes	70 (79%)
	No	19 (21%)
4) Are you aware that it is forbidden to throw cigarette butts on the ground? N (%)	Yes	79 (89%)
	No	10 (11%)
5) Are you aware of the damage caused by cigarette butts in the environment? N (%)	Yes	81 (91%)
	No	8 (9%)
6) Are you aware that it is forbidden to smoke traditional cigarettes and e-cigs in the outdoor areas of schools and universities? N (%)	Yes	50 (56%)
	No	39 (44%)
7) Are you aware that it is forbidden to sell e-cigarettes with nicotine to minors (under 18)? N (%)	Yes	76 (85%)
	No	13 (15%)
8) Are you aware that the University of Milan has anti-smoking regulations? N (%)	Yes	36 (40%)
	No	53 (60%)
9) In your opinion, is the ban on smoking traditional cigarettes in outdoors areas (e.g. courtyards, balconies, porches) of the University complied with? N (%)	Yes	1 (1%)
	No	56 (63%)
	I don't know	32 (36%)
10) In your opinion, is the ban on smoking e-cigarettes in outdoors areas (e.g. courtyards, balconies, porches) of the University complied with? N (%)	Yes	2 (2%)
	No	56 (63%)
	I don't know	31 (35%)
11) What initiatives could the University of Milan undertake to help smokers quit smoking and protect the health of non-smokers? (You can tick more than one box.) N (%)	Informative campaigns on the harm of smoking	43 (48%)
	Greater control over compliance with existing bans	52 (58%)
	Launching specific courses on smoking issues	27 (30%)
	Offering smokers courses to help them quit smoking	58 (65%)
	Other, specify_______________	0
